# Obstetric Violence Against Female Prisoners in Spain: Experiences and Violations of Rights

**DOI:** 10.1155/jp/3728026

**Published:** 2026-05-04

**Authors:** Carmen Meneses-Falcón, Almudena Juárez Rodríguez, Laura María Zanón Bayón-Torres

**Affiliations:** ^1^ Department of Sociology and Social Work, Universidad Pontificia Comillas, Madrid, Spain, upcomillas.es

**Keywords:** female prisoners, gynaecological care, human rights, obstetric violence, prisons, sexual health

## Abstract

**Objective:**

The aim of this study is to explore the conditions of pregnancy and gynaecological care for women in prison in Spain.

**Methods:**

An exploratory qualitative research study was carried out, involving 125 semi‐structured interviews with 79 prison professionals and 46 women prisoners in 16 prisons in Spain. The participants were selected through theoretical sampling. The study was based on a thematic analytical procedure inspired by grounded theory, which produced certain unexpected results that are the focus of this study.

**Results:**

The results of the interviews reveal that obstetric violence (OV) can take place through actors other than healthcare professionals, such as the police force when they guard and transfer pregnant inmates to obstetrics appointments or other inmates who require gynaecological services. During gynaecological examinations and tests, female inmates are kept in handcuffs or restraints and with police officers present in the room, violating their privacy and rights. The responses of gynaecologists to this situation are diverse.

**Conclusion:**

Gynaecologists should be the ones with authority in the medical consultations of female prisoners, and they should not allow the humiliation and violation of the rights of these women, as stated in the United Nations Bangkok Rules. Most female inmates are not dangerous, with non‐aggressive and non‐violent offences, so they would not put the lives of healthcare personnel at risk, and the continuation of this practice is more a question of compliance with protocols than of real risk.

## 1. Introduction

In 2010, the United Nations adopted the Bangkok Rules, which are made up of 70 rules to respond to the needs of women prisoners and to prevent the discrimination they suffer due to their lower numbers in prisons [1]. Rules 6–18 focus on the healthcare they require, including for their children under the age of 3, who, in Spain, are permitted to be with them in mother‐and‐child units. The rules concerned with their sexual and reproductive health are the following: Rule 6(c), referring to reproductive health, which entails the gynaecological and obstetric care that women need whether they are pregnant or not [1]; and Rule 11, which states that only medical professionals should be present when a medical examination takes place, except in extraordinary security circumstances, and if the presence of penitentiary staff is required, these should be female in order to preserve the dignity, intimacy and confidentiality of the prisoner [1]. The other rules in the aforementioned range address mental health, suicide prevention, wrongful use of drugs, sexually transmitted infections and preventative health.

The gynaecological and obstetric care of female prisoners varies greatly depending on the prison [2]. It is better when prisons are exclusively for women than when they are female units in male prisons. It has been shown that female prisoners in the process of pregnancy and childbirth are a vulnerable group [2], with worse health profiles compared with the general female population, due to adverse living conditions before entering prison that do not improve in the prison system [3]. The number of women in prisons has increased in recent decades and, if it continues to do so, more female inmates will face pregnancy in prison, a figure that is currently between 5% and 10% of the population [4].

Spanish prisons, like most prisons in the world, are not prepared to care for women who become pregnant or who enter prison when pregnant or with a baby [5]. There are only two prisons exclusively for women, and they are not equipped for the care of mothers with their children. For this situation, there are six Mother‐and‐Baby Units, two in closed regime and the rest in the community, with 79 mothers in 2022 [6]. However, a pregnancy is monitored in the prison where a woman is admitted, which is close to her and her family’s home, but when she gives birth, she is transferred to a mother‐and‐baby unit [7]. The children can stay with their mothers until they are 3 years old, when they are handed over to their family or to a public administration centre for their care. There were 82 children with their mothers in 2022 [6].

Lack of support and inappropriate treatment during pregnancy and childbirth has been recognised as a form of violence against women and a violation of human rights. Obstetric violence is understood to be disrespectful, offensive or negligent treatment of women by healthcare professionals during pregnancy or childbirth [8], which can result in physical, verbal or psychological violence against them [9–11]. Studies of the general female population and various systematic reviews have highlighted the high prevalence of OV in high‐income countries. Fraser et al. [12] report a prevalence of 45.3%, with the lack of consent from women and the need for information being the most frequently cited aspects. Almost 20% reported verbal abuse and requests for help being ignored by healthcare professionals. Other reviews have pointed out this type of violence with discriminatory and racist connotations towards black women [13], showing differences in age, ethnicity and socio‐economic status. In this regard, the systematic review by Hakimi et al. [11] shows a prevalence of 59%, depending on the country, and found a correlation between the high economic status of women and the low risk of gender‐based violence (GBV), indicating that it is also related to better access to health resources. In other words, GBV decreases as socioeconomic status increases.

The definition of OV seems only to include healthcare personnel [14]. However, the present study highlights other social actors and contexts that may be involved in this violence that we could call obstetric and institutional during the process of pregnancy or childbirth, and also in preventative sexual or reproductive health check‐ups. We refer to the prison context and the police custody that women undergo during gynaecological and obstetric check‐ups. This aspect is rarely referenced in academic literature. Therefore, the concept should include other social agents that intervene in the maintenance of obstetric, reproductive and gynaecological health. It has been found that in some countries, female prisoners are shackled or put in leg irons during transport to hospital, gynaecological examinations and childbirth [5]. This form of institutional violence contradicts the spirit and stipulations of the Bangkok Rules.

Some studies on women in prison have mentioned aspects that can be linked to OV. In Abbott’s investigation [15] in two English prisons, she highlighted the humiliation experienced by pregnant inmates when they were handcuffed during obstetrics services, citing the risk of escape. The prisoners pointed out that being pregnant or about to give birth were not the best circumstances for escaping, and that handcuffs were a way of asserting power and control over them in public healthcare spaces. In their paper, Brawley and Kurnat‐Thoma [16] highlight the use of shackles on female inmates as a security measure and to prevent escape, but also to follow protocol, in some North American prisons. They point out that there are no published reports of escape attempts by women incarcerated without shackles during labour, delivery or the postpartum period, in addition to the fact that the majority of incarcerated women are non‐violent offenders. They also add that the reason for the use of restraints is to follow legal protocol rather than a real threat.

Jubany‐Roig and Masso‐Guijarro [6], in their study with 30 Spanish female prisoners, highlight the medical recommendation to the prisoners to stop breastfeeding, whether or not there was a justified medical or health reason to do so. They point out that in a hostile environment such as prison there are no strategies for the promotion, protection and support of breastfeeding. They even described humiliating and degrading situations at the hands of the police who were guarding them, pressuring them to abruptly stop breastfeeding their babies.

Other studies have shown that, in some prisons, during the transfer to hospital for check‐ups or childbirth, the prisoners are kept in shackles or chains throughout the journey, as well as during the recovery period, whether in prison or hospital [17]. These aspects have been highlighted by Amnesty International [18] and in Spain by the Spanish Ombudsman [19], due to the police presence at childbirth, as a violation of the right to privacy. Imprisonment increases women’s vulnerability and their pregnancy and childbirth process as well [3].

The negative consequences of OV on the female population in general have been demonstrated in the literature. Depression and anxiety have been found to be high (51% and 57%, respectively) among pregnant prisoners [20], as well as low emotional adjustment and interaction with the baby. A traumatic experience during childbirth has been linked to a lower level of attachment to the newborn, affecting the mother–child relationship [21]. Stress, depression, post‐traumatic stress and difficulties in the child’s development have also been described when the treatment throughout this process is inadequate [22]. However, the limited availability of databases on the outcomes and care of pregnancy in incarcerated women has also been highlighted [23]. The use of handcuffs or shackles on pregnant women increases the risk of falls, which in turn increases the risk of placental abruption, haemorrhage and foetal death, due to a reduced ability to break the fall [17]. The risk of a thromboembolic event is high during pregnancy and the postpartum period, and limited mobility with shackles increases this risk and the decrease in mobility reduces the possibility of a spontaneous vaginal delivery because it interferes with the progression of labour [17].

In view of the above, this study has the following objectives, after obtaining emergent results by examining the variables of reproductive health: (1) To explore the conditions of reproductive health care for incarcerated women in Spain. (2) To examine compliance with the Bangkok Rules on reproductive health. (3) To identify the care needs of female prisoners in obstetric and gynaecological check‐ups.

## 2. Methods

### 2.1. Design and Participants

A descriptive exploratory study was carried out with a qualitative approach, using semi‐structured interviews. A total of 125 interviews were conducted in 16 prisons in Spain: three prisons were women only, the rest were male prisons with populations between 500 and over 1000 inmates in 5–10 male units, and in these prisons there were between 50 and 200 women inmates, in one or two female units. Eighty of the 125 interviews were with professionals working in the prisons and 45 were with female inmates. Theoretical sampling was applied based on predefined categories [24]. The prisoners were selected by prison staff based on a series of criteria (age, nationality, whether or not they were mothers, length of time in prison, whether or not they participated in prison activities). The professionals had to meet the following criteria: type of professional (health, social work, psychologist, educator and prison officer), a minimum of 3 years at the prison where the interview was conducted, and at least 2 years′ experience working with women. The interviews began in January 2023 and ended in July 2023. Authorisation to carry out the interviews was obtained from each respective penal institution.

### 2.2. Data Collection and Information Sources

The interviews were carried out by the research team, who travelled to each prison, and took place in private offices located at the entrance to the prison wings, where confidentiality was guaranteed. They lasted between 30 and 60 min and were audio recorded with the informants’ consent. When they felt uncomfortable with the recording, notes were taken as exhaustively as possible. An interview script was prepared with different topics following the Bangkok Rules (before entering prison, imprisonment, prison classification and unit, physical and mental healthcare, participation in educational and recreational activities, treatment from staff, searches, punishments and means of coercion, relationship with family and children, abuse received, prison regime and relationship with prison personnel), but this paper only addresses and analyses gynaecological care.

### 2.3. Analysis

A descriptive, categorical and interpretative analysis of the interviews was carried out, avoiding details that could lead to the identification of each participant. Analytical categories were developed that enabled the coding of all the interviews to later extract those segments that were required for the analysis and interpretation of the proposed objectives. The study was based on a thematic analytical procedure [25] inspired by grounded theory [24], which produced certain unexpected results that are the focus of this study. Once the interviews had been conducted, recorded and transcribed verbatim, the grounded theory model, ‘which combines, through a constant comparative analytical procedure, explicit coding and theory development’, was applied to the interviews carried out in different prisons [26]. First, a general reading of the transcripts was carried out. This was followed by a review of the emergent themes, of which those related to physical, psychological and gynaecological health are relevant for this article. Five dimensions were formulated based on the Bangkok Rules: Health, Prison System, Security, Children and Family, and Minorities. From the first three, themes, categories, and subcategories emerged, from whose interrelationship the emerging results were produced inductively. From Health came *gynaecological care* and *pregnancy*; from the Prison System came *type of prison* (women‐only or units in men’s prisons); and from Security came *discipline*, *abuse and violence* and *mistreatment.* Finally, an identification of interrelated themes was made. The categories of analysis were established and triangulated independently by members of the research team to increase the reliability and validity of the analytical process. This entire process was carried out with the support of NVivo 12 qualitative analysis software.

**Figure 1:**
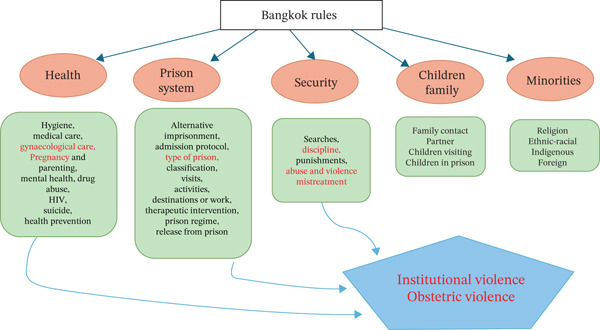
Thematic map.

The map below (Figure 1) shows the process of construction of the categories and subcategories of analysis that led to the emergence of OV.

The main categories arise out of the Bangkok Rules, out of which other thematic aspects also emerge, also contained in the Rules, with their interrelation leading to OV. Thus, gynaecological care is related to the prison type and to the security setup in prisons—with higher security for male than female inmates—and to the healthcare categories. Medical care is one of the important pillars of the prison system. However, security aspects can lead to mistreatment and abuse of inmates, due to various circumstances, which have been collected in the women’s narratives.

### 2.4. Ethical Considerations

The project was approved by the university’s ethics committee. The research team ensured compliance with all the requirements: voluntary participation, anonymity and confidentiality. Prior to conducting the interview, the members of the research team presented an informed consent form to all the interviewees. This consent form stated that the interviewees were participating voluntarily and that the interviewers would not reveal any information that could identify them. The inmates were assured that their participation in the research would not affect their prison sentence and that they could end the interview at any time or refuse to answer any questions that made them feel uncomfortable.

## 3. Results

### 3.1. Characteristics of Participants Interviewees

The participants were the following: 16 members of prison management teams (director or deputy director), 13 social workers, 10 psychologists, 8 educators, 19 interviews with female prison officers, 1 lawyer and 5 doctors, in an age range from 30 to 64 years old, with more than 2 years of experience in the prison where they were at the time of the interview, and who had worked with women in prison. In this investigation, social workers, psychologists, social educators and health workers made reference to the subject under study. The rest were unaware of the care received by the inmates when they left the prison and went to the gynaecology and obstetrics services.

The 46 female inmates who participated in the study were aged between 23 and 52; 90% of them were mothers; three were of Romani ethnicity; 24 were convicted and the rest were on remand; 12 had drug problems; 5 reported being abused by their partner; 3 had a recognised mental disability; and 2 were undergoing psychiatric treatment.

### 3.2. Gynaecological Care in Prison

In most Spanish prisons, particularly when the number of women is small, female inmates are taken outside the prison to a gynaecology specialist for a medical check‐up, or to the obstetrics department if they are pregnant. Only in women’s prisons is a gynaecologist regularly sent to carry out check‐ups. However, diagnostic tests in gynaecology or obstetrics are carried out outside the prison in both cases, either in specialised medical centres or in hospitals. The inmates complained that the medical examinations were not annual and that in some prisons they were not attended to at all.

*It’s true that there was no gynaecologist, midwife or anything here. And when we asked, they said no, I think they are looking into how we can be seen. They take us to the hospital*. (PV17. Inmate, 50 years old, northern Spain).

*They did one here, when I was 50. Now I’m 51. I told them I have a problem: I had a warning of uterine cancer during my youngest daughter’s pregnancy. I would like them to do more for me, at least one a year because I was already going through the menopause. I’m waiting because they told me they were going to call me in for a mammogram. I’m going to be 52 and I’m still waiting. If you get sick at the weekend, they will not bring you down. (*PV15. Inmate, 51 years old, northern Spain).

However, despite the fact that the legislation states that female prisoners have the right to check‐ups related to their gender, the reality is that in many Spanish prisons, these check‐ups are carried out with a long delay except in cases of pregnancy, particularly in prisons that are for men with one or two women’s wings. In the interviews, we even learned that some prisoners who had been in prison for more than 3 years had never been seen by a gynaecologist.

The first problem that threatens the dignity of the inmates is the transfer to hospital for the gynaecological check‐up.

*The police take me, I’m handcuffed. I tell them that I should not have to be handcuffed because I’m pregnant. I always made medical visits with the police and in handcuffs. All men. It’s forbidden. Being pregnant I should go in an ambulance. All the visits in xx were in a police car and in handcuffs. Never here. When I arrived here in Madrid, to be honest, yes, always in an ambulance.* (E74. Inmate, 23 years old, central Spain).

One of the only two prisons that are exclusively for women is in Madrid, with several units and services exclusively for the inmates. In these cases, the gynaecologist visits the prison and the inmates are only taken out to carry out necessary tests. The police in charge of these transfers are responsible for the transport and the criteria for the use of handcuffs are not clear, because while some police officers do not use them, others always do. The second problem occurs when they arrive at the gynaecologist’s office and two situations arise: they remain handcuffed and the police stay inside the doctor’s office.

*If I go to the gynaecologist, sometimes, if I do not have a woman, a man goes, but they came in, but he pulled the curtain and they stood with their backs turned.* (E107. Inmate, 50 years old, northern Spain).

*Yes… I felt a bit uncomfortable there because, I do not know, I’m like that, it’s not because of my culture or anything, but when, for example, you are lying down, your breasts are more visible. And I did not like him being there. I would have liked him to be at the door.* (E91. Inmate, 22 years old, central Spain).

*She had a gynaecological appointment, and they did not remove her handcuffs, the Guardia Civil officers go into the doctor’s office with you. They stay with you during the appointment. My cellmate was very upset because she had to undress, she could not take off her clothes because she was handcuffed, a nurse had to take off her trousers and she was on her period, the police were at the door. … sometimes they stay at the door… and if you have to undress, they close the door and stay there. My friend was upset because she could not do anything, she was handcuffed and they had to take off her clothes, her panties, her sanitary towel, the police were there, and she was extremely uncomfortable.* (E89. Inmate, 28 years, central Spain).

*I felt awful. If you have a doctor’s appointment they are there, they do not give you any privacy. That treatment is very bad. At the gynaecologist, even if they close the curtain you know they are there. If a girl goes with you, they handcuff your hands in the office, they do not take them off. It depends on each police officer. The route is very windy and the handcuffs are behind you. You feel terrible. Even if the woman goes in, the man goes in too. They are nice, but they make you feel uncomfortable. You have no privacy*. (PV16. Inmate, 39 years old, northern Spain).

When the research team asked prison staff about this police procedure, which violates the dignity and privacy of the inmates, there were a variety of responses. Some were unaware of these facts, while others were aware but said that the police were solely responsible and that prison staff had no authority over transfers.

*We can find ourselves in a situation where everything is going smoothly and there are no problems, or even in some cases where the hospital social workers have notified the prison, the social worker, that certain guards are present at the inmates’ gynaecological examinations. This situation should not occur, but there is no prohibition in this respect to prevent it from happening.* (E16. Social worker, southern Spain).

*No one has complained to me about that and, besides, I am a fairly open person and if an inmate comes to me and says that, it automatically becomes a complaint, you have to put it in writing and bring it to the attention of the management. Full stop.* (E102, Social Educator, northern Spain).

*It’s not up to the Prison Service, it’s up to the security forces. They’re the ones who decide whether there are handcuffs or not.* (E106. Psychologist, northern Spain).

Regarding the gynaecologists’ behaviour in the consultation room, we found various actions.

*I went to the gynaecologist and the doctor herself had the handcuffs removed, of course.* (E108. Inmate, 41 years old, northern Spain).

*When I was inside the consultation room, yes, because the gynaecologist asked: ‘you can take off the shackles now’.* (E109. Inmate, 35 years old, northern Spain).

*The doctor told them to get them out, to take off my handcuffs, but they said no.* (E101. Inmate, 29 years old, southern Spain).

Medical personnel do not act in the same way. In some cases, they ask the police to remove the handcuffs and stay outside, and in others, they do not get involved in the matter. It is possible that a gynaecologist’s demand has an effect on the police, given that inside the clinic it is the doctor who has the authority.

Security is the overriding theme in the prison system, and all other actions are contingent to it. At the same time, the medical care of prisoners is another essential aspect, because any harm to inmates’ health is negligence and the responsibility of the state. In this sense the two systems, and their own practices, interact. The healthcare professionals who work in prisons follow the established protocols and are more accustomed to the security measures required, but this is not the case with health professionals outside the prison system, whose priority is the good care of the people they attend to. For this reason, the gynaecologists can find themselves in an uncertain situation when security unjustifiably overrides their healthcare standards in their offices. We have referenced the assertion that female inmates are less dangerous, show less potential for conflict, and whose crimes tend to be less serious, with the result that the severity of security against the risk of violence, flight or conflict is much less than in men’s prisons. The confusion may be greater when the officers or staff do not always act in the same way and follow different protocols: for example, if the female inmates come from a women‐only prison, with lower levels of security, or if they are in male or mixed prisons, where the measures are stricter or even severe. Greater knowledge and empowerment of these professionals could limit the unjustified actions of security officers and guarantee the dignity and respect of their patients during gynaecological care.

**Figure 2:**
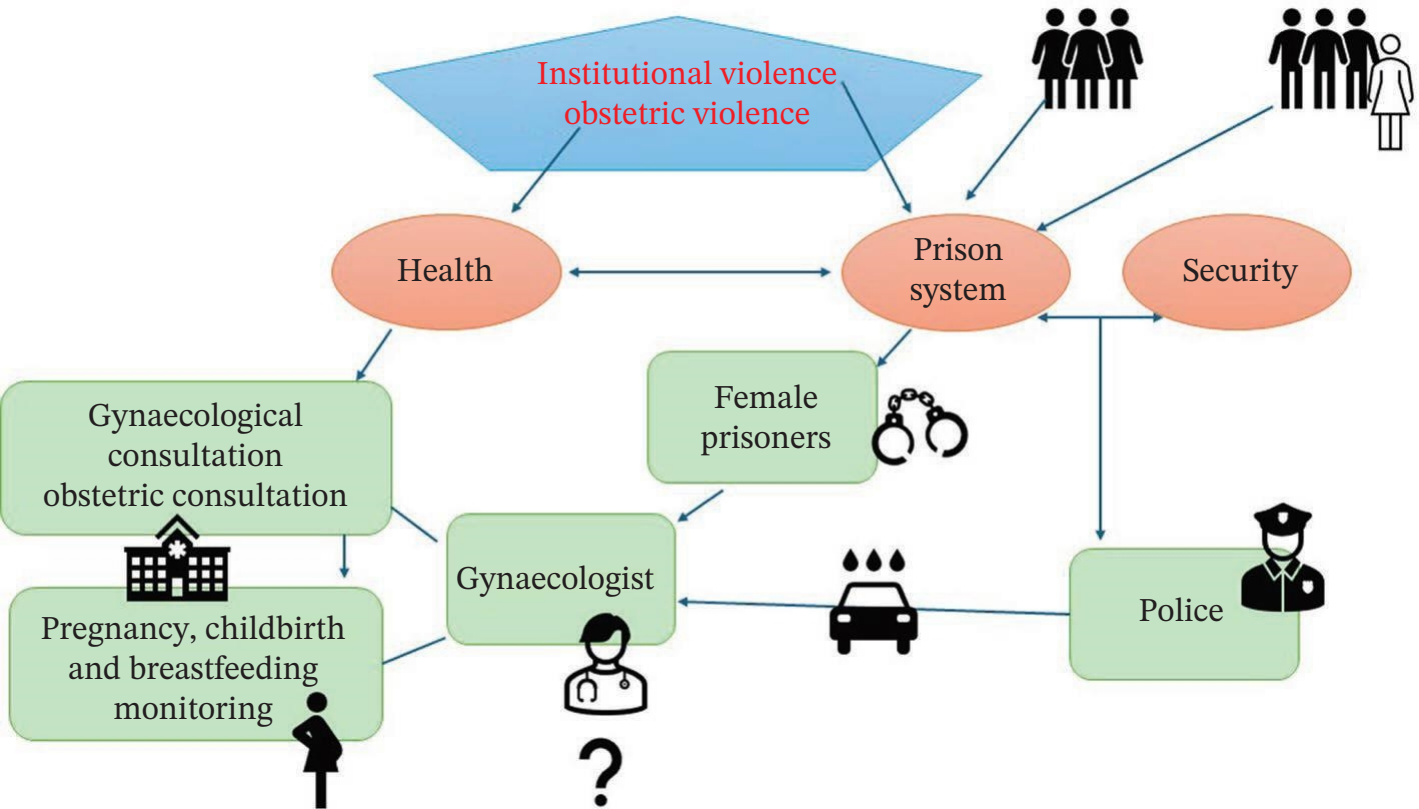
Summary of key findings.

In the above diagram (Figure 2), we summarise the main findings, showing how institutional OV emerges at the intersection of two state institutions: the healthcare system and the prison system. Within prisons, this form of violence appears to be more prevalent in mixed‐gender facilities and less frequent in women‐only prisons. The map also depicts the key actors involved, as well as healthcare staff’s reported uncertainty when they encounter police officers accompanying female prisoners into consultations, without removing handcuffs and without leaving the room during the appointment.

## 4. Discussion

This work has highlighted how OV can be carried out by other social actors, such as the police, and in other contexts, such as prisons, since it is not only health personnel, in hospitals or health centres, who are involved. Female prisoners who attend gynaecological and obstetric services may find themselves in situations, brought about by police officers, of inappropriate treatment, with little support, in humiliating circumstances and with a lack of information and consent regarding their sexual and reproductive health. The definition that studies make of OV can be applied to female prisoners, as they often suffer institutional violence when they go for gynaecological check‐ups, are pregnant or are about to give birth. The dimensions covered by OV refer to the violation of women’s autonomy due to lack of information or consent, their dignity and their rights, with various indicators involved [27]. Of these, our results highlight degrading treatment and excessive control that prevents autonomy as a patient in gynaecological consultations through being handcuffed, which was forced upon the participants without objective reasons for any of them. This type of violence can be classified as institutional violence, as referred to in other studies taking Foucault’s perspective, in relation to interactions between individuals within state institutions. In this case, these are two institutions that interact in women’s health: the health system and the prison system, one strictly female (gynaecology) and the other mainly male, as highlighted by feminist criminology [28].

The results obtained in this study are consistent with other studies on prisons when women are pregnant, breastfeeding their babies or who have to go to the gynaecologist as a preventative measure [6, 15, 17]. Abbott [15] found situations similar to those found in this study with female prisoners. Abbott proposes the concept of *institutional ignominy* to describe the experience of English women when they went to hospital and were displayed as pregnant prisoners, reinforcing the power and control of the prison institution with the presence of handcuffs or shackles in the healthcare context.

The need for preventative healthcare assistance specific to women is reflected in the Bangkok Rules, specifically Rule 18. Female prisoners are deprived of personal liberty but this does not mean deprivation of their right to health, regardless of whether they are pregnant or not. In this study, only a third of the surveyed inmates had undergone a gynaecological examination in the past year and given that many of them fall within the age range of 40–50 years old, which is when menopause can begin, annual gynaecological check‐ups should take place for a larger proportion of prisoners.

The transfer of prisoners with handcuffs, shackles and restraints to the hospital or delivery room, and their not being removed in the consultation or delivery area, is an affront to the dignity of women. Although the suppression of this practice is stipulated in many laws, it is not uniformly applied. This has been noted in the United States [16, 23], England [15] and Spain, where the number of women in prison is smaller, as well as the number of pregnant women. It is also reflected in the Bangkok Rules, specifically Rule 24 regarding means of coercion, which pregnant women should be exempt from at all times. These degrading transfers lead to the increase in psychological distress and exacerbation of mental health problems for these women, while shackles or chains can pose a risk to their health, and violate human rights and national and international obstetric and gynaecological standards [23]. Some of the interviewees were aware that their rights were being violated but could not do anything about it. The relationships of subordination, control and submission to police authority in institutions such as prisons do not allow female prisoners to demand that their rights be upheld. The main psychological and physical consequences referenced in the literature were not mentioned in the participants’ interviews. However, it was noted that the embarrassment and shame experienced during the gynaecological consultation led to a reluctance to attend further appointments, with the risk that the absence of preventative check‐ups could pose to their reproductive health.

It must be emphasised that, in medical consultations, it is denigrating for prison staff or police to be present, especially when they are men, as the previous studies cited have highlighted. Medical examinations or assistance should only be provided by healthcare personnel, and only in cases of risk of escape should the presence of a female prison staff member be admitted, but not the police, according to Rule 11 of the Bangkok Rules. However, the flight risk for women is low and even less so when they are pregnant or about to give birth. Spanish female prisoners are generally not aggressive or violent [29], particularly if they are pregnant, and it makes no sense to keep them shackled during a medical consultation. The recidivism rate of Spanish female prisoners (13.5%) is lower than that of men (20.5%) [30], and most crimes committed are non‐violent and without bodily harm. These practices degrade the dignity of female prisoners and violate their human rights.

Spanish prison legislation does not allow for the application of isolation as punishment for pregnant female prisoners or those with infants. It is noteworthy that, although it has happened to a minority, the inmates interviewed have reported being placed in isolation during this perinatal or postnatal period, generally due to punishment for non‐compliance with rules. This data should be studied in depth because it represents a very serious violation of women’s and children’s rights. Additionally, it has been questioned whether pregnant women should serve their sentence in a closed prison and whether the prison is responsible for monitoring their pregnancy and babies. Alternative measures to the prison or external units could be drawn up, in which community gynaecology and paediatric services could attend to these women and their children [4].

The experience of pregnancy in prison is more stressful and dangerous than outside [6] because the future of a prisoner’s baby will be uncertain (she remains with it until age 3 or must separate from it at birth and hand it over to her family or authorities). The identity of the pregnant prisoner was dichotomised by a double status: that of a pregnant woman and the label of prisoner/criminal [31]. Furthermore, it has been noted that pregnant prisoners or those with young children are a vulnerable group because this situation is compounded by factors such as poverty, addiction, early age, GBV, mental health and they require very specific attention to these vulnerabilities, which are not provided in most prisons [32].

Prisoners are deprived of liberty but not of their rights. Their reproductive health check‐ups are insufficient, either due to a lack of specialist medical personnel in the prison or difficulties with transporting them outside for medical care. During these transfers to the hospital, discriminatory and dehumanising forms of treatment often occur, sometimes stemming from prejudice against women prisoners [15, 16]. The primacy of surveillance and security measures, requiring pregnant women to be shackled or subjected to other forms of restraint, stands out as the most extreme and visible form of detected violence, both obstetric and institutional.

Gynaecologists, as this work has documented, often assert their expertise and authority in medical consultations, especially when prisoners are pregnant, to prevent the use of shackles or other restraints. However, their opinion or demands are not always followed. In this regard, the authority of healthcare personnel should prevail in medical consultations unless there is a justifiable risk of escape or aggression by the prisoner. This aspect should be considered by prison institutions in coordination with health centres and hospitals.

There are some considerations or limitations to keep in mind about this study. Because this was an exploratory work, we were unable to go into details and depth on different aspects of reproductive health for female prisoners. Nevertheless, institutional violence emerged as a secondary consideration because it was an unexpected result that emerged from analysing the qualitative interviews. This emergent nature has precluded us from delving deeper into many aspects related to pregnancy development and childbirth. Moreover, it is an exploratory qualitative study, so its results cannot be generalised to the entire female prison population. The selection of inmates was carried out with the guidance of prison staff, which may have introduced selection bias and affected the diversity of experiences collected. Although selection by the research team was not possible, the researchers are experts in interviewing vulnerable groups and this made it possible to establish trust and a bond for the communication and sharing of the infringement of inmates’ rights. This bond that was established with many of the participant inmates lessened the hierarchical relations that can occur in the context of interviews between interviewee and interviewer. Although there are reports of shame and reluctance to attend new medical consultations, the study did not specifically assess broader psychological or physical consequences, so these should be interpreted with caution. This means that in future studies, we should apply greater detail to the entire process of pregnant women and gynaecological health for female prisoners, as well as the prevalence of these actions on female prisoners in Spanish and European prisons.

Through sociology and criminology, institutional violence in prison can be understood as a structural effect of the logic of total institutions as described by Goffman: closed spaces in which the separation between daily life, vigilance, discipline and control of the body drastically reduces the autonomy of interned people and normalises practices that, outside of that framework, would be clearly harmful or degrading. In this sense, the use of handcuffs during transfers to hospital, childbirth or gynaecological check‐ups does not only constitute a one‐off security decision but an expression of what the criminological literature identifies as institutional violence and ‘excessive guarding’: the systematic subordination of care to the logic of security. This tension can become intensified in the case of female prisoners because the reproductive body is situated at the intersection between punishment, moral control and risk management, converting obstetric care into a space of dispute between medical knowledge and police authority. Thus, prison rationality is not suspended in the clinical space but colonises it: the care criterion of healthcare staff can be ousted by the coercive power of the security forces, even when there is no individual indication of danger. Furthermore, the differences between women‐only prisons and women’s units inside male prisons can be interpreted in the light of the sociology of prison organisation and geography: when women occupy residual spaces within institutions that are materially and normatively designed for men, their healthcare tends to depend on more precarious, less specialised routes that belong more to masculinised guarding routines, which can favour a higher prevalence of restraining practices and bodily dispossession. The architecture, spatial distribution, availability of staff and organising culture are not, therefore, neutral variables, but conditions that shape the intensity with which the punishment penetrates the caregiving sphere and that shed light on how OV in prisons is not an accident but a specific manifestation of institutional violence exercised on female bodies in prison.

### 4.1. Implications for Practice

Training and sensitisation of healthcare, prison and police personnel regarding this reality is essential to ensure greater involvement from both prison institutions and the healthcare system, and to eradicate these practices. Furthermore, inter‐institutional coordination is shown to be a crucial element to ensure that transfers and medical care do not violate the rights of female prisoners.

For pregnant women or those with children in their care, it would be necessary to consider alternative measures to imprisonment that can help reduce stress and risks associated with pregnancy and childbirth in a situation of strict deprivation of liberty, reducing post‐traumatic stress, depression and difficulties in maternal‐filial relationships.

## 5. Conclusions

In conclusion, the OV suffered by women in prison represents a grave violation of human rights and directly contravenes what is established in the Bangkok Rules of the United Nations, which promote a gender‐sensitive approach to treating female prisoners. These rules emphasise the importance of ensuring adequate healthcare services, including gynaecological and obstetric care, as well as respecting dignity and providing humane treatment during pregnancy, childbirth, and postpartum. The persistence of neglectful or abusive practices in the prison context not only perpetuates structural inequality but also ignores the international normative framework designed to protect vulnerable populations.

It is essential to continue researching and monitoring gynaecological and obstetric care in prison contexts, with the aim of identifying areas for improvement and evaluating the impact of implemented interventions. Future research lines could include longitudinal and comparative studies with other countries, with the goal of identifying and adapting best practices. Additionally, it is crucial to delve deeper into the psychological impact of OV on women deprived of their liberty, considering consequences such as post‐traumatic stress, depression and difficulties in maternal–filial relationships.

## Funding

This study was supported by Ministerio de Ciencia, Innovación y Universidades, 10.13039/100014440, PID2022‐140134OB‐I00.

## Conflicts of Interest

The authors declare no conflicts of interest.

## Data Availability

The data that support the findings of this study are available on request from the corresponding author. The data are not publicly available due to privacy or ethical restrictions.
